# Salivary Metal Ions as Potential Biomarkers for Diabetes: An Observational Study

**DOI:** 10.1155/ijod/9224685

**Published:** 2026-01-16

**Authors:** Zihan Ding, Jieyu Ming, Huajun Dai, Liling Chen, Bing Guo, Weiqi Li, Xing Zhao, Hang Zhao, Hao Xu

**Affiliations:** ^1^ State Key Laboratory of Oral Diseases and National Center for Stomatology and National Clinical Research Center for Oral Diseases and Research Unit of Oral Carcinogenesis and Management and Chinese Academy of Medical Sciences, West China Hospital of Stomatology, Sichuan University, Chengdu, 610041, Sichuan, China, scu.edu.cn; ^2^ Chengdu Institute of Food Inspection, Chengdu, 611130, Sichuan, China; ^3^ Chongqing Municipal Center for Disease Control and Prevention, Chongqing, 402100, China; ^4^ West China School of Public Health and West China Fourth Hospital, Sichuan University, Chengdu, 610041, Sichuan, China, scu.edu.cn

**Keywords:** biomarker, diabetes, dose–response relationship, propensity scores matching, restricted cubic splines, saliva metal ions

## Abstract

**Objective:**

Saliva is an important body fluid that reflects systemic health. This study is a cross‐sectional study aimed at exploring the relationship between salivary metal ion concentrations and diabetes risk.

**Methods:**

From the China Multiethnic Cohort (CMEC), 912 eligible participants were enrolled, and their saliva samples were collected. Salivary metal ion concentrations were measured using an inductively coupled plasma mass spectrometer and inductively coupled plasma optical emission spectrometry (ICP‐OES). Logistic regression and propensity score matching (PSM) were used to examine the association between salivary metal ions and diabetes. Additionally, restricted cubic spline (RCS) analysis was used to explore the dose–response relationship between specific metal ions and diabetes risk.

**Results:**

This research initially revealed a notable nonlinear dose–response association between the levels of iron in saliva and diabetes risk, and it also identified a significant relationship between salivary manganese and diabetes.

**Conclusions:**

Significant associations were identified between salivary concentrations of iron and manganese and diabetes risk. Monitoring salivary metal ion levels may aid in the prevention and management of diabetes.

**Clinical Significance:**

The findings suggest that analyzing salivary metal ions could serve as a noninvasive tool for assessing diabetes risk, offering a valuable approach for early intervention and management.

## 1. Introduction

Diabetes is a metabolic disease characterized by high blood sugar levels and represents one of the most common and rapidly increasing diseases globally [1]. Diabetes often leads to cardiovascular, renal, retinal, and neurological complications [2, 3]. These conditions can severely impact health and finances and may even be life‐threatening. Diabetes is a complex disease influenced by genetics, environment, and lifestyle factors [4]. Thus, prevention and early detection are crucial for managing diabetes and preventing its complications. Currently, the detection and daily management of diabetes mellitus mainly relies on the collection of venous blood or fingertip blood. Although portable blood glucose meters have facilitated patients’ daily blood glucose testing, this invasive operation will bring psychological burdens to patients, which in turn will reduce their compliance. In addition, the requirements for diabetes testing indicators are complicated. Taking fasting blood glucose (FBG) and oral glucose tolerance test (OGTT) as an example, the collection of blood samples has strict requirements on the fasting status of patients and the sampling interval. Although glycated hemoglobin (HbA1c) can reflect the blood glucose level over a long period of time, it cannot accurately reflect the blood glucose level under some special circumstances, such as the presence of diseases that affect hemoglobin metabolism, and therefore cannot be used as a screening indicator for diabetes [5, 6]. Therefore, the development of a noninvasive and simple monitoring indicator is crucial for the effective management and real‐time monitoring of diabetes.

Saliva is a vital biological fluid secreted by the salivary glands and plays a key role in maintaining oral health [7]. The composition of saliva is closely linked to the body’s physiological state [8], supporting frequent daily sampling to capture dynamic metabolic changes. Due to its simple collection process, which requires no fasting or anticoagulation treatment and involves easy storage conditions, saliva is considered especially suitable for community screening, children, the elderly, and patients with low compliance [9, 10]. Given its noninvasiveness, convenience, real‐time capability, and broad accessibility, saliva holds great potential as a biomarker repository for disease diagnosis and monitoring [11]. During the COVID‐19 pandemic, saliva testing became widely recognized as an efficient and convenient tool for virus detection [12]. Salivary biomarkers, such as DNA, RNA, proteins, metabolites, and microbiota, have been identified [13–17] and can be applied to the early diagnosis and monitoring of oral diseases, metabolic disorders, neurodegenerative diseases, and gastrointestinal conditions [18, 19]. As a chronic metabolic disorder, diabetes often affects the secretion and synthesis of saliva [20]. Research has indicated that alterations in salivary concentrations of amylase, resistin, and glucose may serve as potential biomarkers for diabetes diagnosis and monitoring [21–23]. However, improving the accuracy of noninvasive diabetes monitoring remains a significant challenge [24] highlighting the need for more reliable biomarkers.

Metal ions are essential in maintaining cell functionality and supporting various physiological processes, including catalysis, metabolism, and signal transduction [25–27]. Research has shown that imbalances in metal ion homeostasis are linked to the mechanisms of various diseases, such as metabolic, neurological, cardiovascular, and oncological disorders [28–30]. It is now established that metabolic abnormalities in zinc, magnesium, iron, copper, and manganese are closely associated with diabetes and its complications [21, 31, 32]. The oral cavity is a key pathway for the intake of exogenous metal ions, primarily from the diet and the release of ions from dental materials [33, 34]. Research has indicated that saliva contains various metal ions. These metal ions enter saliva mainly through active transport and passive diffusion from plasma, with their concentrations considered to reflect systemic metal ion levels [35]. Furthermore, changes in the concentration of metal ions in saliva can reflect real‐time physiological states [14, 36]. This characteristic is particularly valuable for diabetic patients who require long‐term blood glucose monitoring [5]. Therefore, metal ions in saliva are promising as potential biomarkers for diabetes. However, the detection of metal ions is not currently included in clinical diagnosis guidelines for diabetes [37]. Based on this gap, noninvasive detection of diabetes is anticipated to be developed in the future, offering a more efficient and convenient approach for daily management and real‐time monitoring.

Inductively coupled plasma mass spectrometry (ICP‐MS) and ICP optical emission spectrometry (ICP‐OES) are valued for quantifying metal ion concentrations. ICP‐MS offers detection limits from pg/L to ng/L for trace elements like Pb, As, and Cd. ICP‐OES provides a linear range from μg/L to mg/L with strong resistance to matrix interference for major elements such as K, Ca, and Mg [38–41]. Logistic regression is noted for quantifying associations and supporting variable selection [42]. Propensity score matching (PSM) enhances accuracy by reducing confounding through variable matching [43]. Restricted cubic spline (RCS) analysis preserves continuity to explore nonlinear dose–response relationships [44]. These methods are increasingly applied to investigate the prediction of diabetes risk [45–47].

In conclusion, we conducted a cross‐sectional study aimed at exploring the association between salivary metal ion concentrations and diabetes risk. The objective of our study is to identify metal ions that are significantly associated with diabetes risk and further analyze the relationships between them, providing a theoretical foundation for the application of salivary metal ion testing in clinical diabetes diagnosis and monitoring.

## 2. Methods

### 2.1. Population

The study participants were sourced from the China Multiethnic Cohort (CMEC), a prospective community‐based study in Southwest China designed to investigate the prevalence, risk factors, and associated conditions of noncommunicable diseases among ethnic minorities [48]. Participants were recruited using a multistage, stratified cluster sampling approach. The study population was drawn from five provinces in Southwest China (Sichuan, Chongqing, Yunnan, Guizhou, and Tibet) and included ethnic minorities such as Tibetan, Yi, Miao, Bai, Bouyei, and Dong, as well as the Han ethnic group. In total, 99,556 participants were recruited to complete health screenings and questionnaires between May 2018 and September 2019 in the CMEC study. After excluding those without saliva samples (*n* = 98,554) and those whose saliva samples did not meet the quality requirements for metal ion detection (*n* = 90, including samples with a volume less than 1 mL or containing solid particles), we enrolled 912 participants, including 138 individuals with diabetes. All participants provided signed informed consent. The study protocol was approved by the Medical Ethics Review Committee of Sichuan University (K2016038).

### 2.2. Sample Collection and Storage

Passive salivation was used to collect saliva in this study. In the absence of external stimuli, saliva was allowed to naturally flow. 2 mL of saliva was collected using a sterile collection tube. Participants were instructed to refrain from eating, drinking, or engaging in any oral hygiene practices for at least 30 min prior to collection. This method is suitable for analyzing saliva under normal physiological conditions [23].

Regarding saliva storage, the samples were immediately placed in an icebox after collection and transported to the laboratory within 24 h. The samples were then stored at −80°C.

### 2.3. Detection of Metal Ions in Saliva Samples

The concentrations of 19 metals (K、Ca、Na、Al、Mg、Pb、Li、Cr、Ni、As、Se、Cd、Sb、Mn、Fe、Cu、Zn、Sr、Ba) and one metalloid (Sn) were analyzed in the saliva samples of all study participants. During the sample preparation, nitric acid was added to the samples, which were then digested using a microwave digestion system (Multiwave PRO; Anton Paar, Austria). The sample pretreatment was conducted using a microwave‐assisted nitric acid digestion method. A well‐mixed sample was weighed into a digestion vessel, and 6 mL of pure nitric acid was added [49]. After resting for 1 h, the vessel lid was tightened, and the digestion vessel was placed in a microwave digestion system (Multiwave PRO; Anton Paar, Austria) for digestion (Table S1). After cooling, gas was slowly released, and the solution was heated at 100°C for 30 min to remove excess acid. The digested solution was then transferred to 25 mL (for ICP‐MS) and 10 mL (for ICP‐OES) volumetric flasks, diluted with ultrapure water, mixed thoroughly, and prepared for use. The concentrations of Pb, As, Cd, Sb, Mn, Fe, Cu, Zn, Sr, Ba, and Sn were measured using ICP‐MS (iCAP RQ; Thermo Fisher Scientific, USA), while K, Ca, Na, Al, Mg, Li, Cr, Ni, and Se were detected using ICP‐OES (PE 8300; Perkin Elmer, USA) for quantitative analysis of metal ions. All procedures strictly followed the manufacturer’s recommended protocols (Table S2,S3).

### 2.4. Covariates

This study’s covariates included: age, gender, education level, smoking status, alcohol intake, BMI, hypertension (HTN), and hyperlipidemia (HPL). Gender was coded as 0 for male and one for female in the study dataset. Education level was classified based on the highest degree obtained, including categories such as none, primary, junior high, high school, college, and university or above. Smoking status was divided into never smokers, current smokers, and former smokers. Participants were assigned to groups of nondrinkers, light/moderate drinkers, and heavier drinkers according to their alcohol intake. According to World Health Organization (WHO) guidelines, the body mass index (BMI, measured in kg/m^2^) serves as an indicator of obesity [44]. HTN was identified when systolic blood pressure (SBP) ≥ 140 mmHg and/or diastolic blood pressure (DBP) ≥ 90 mmHg [50]. A diagnosis of HPL was confirmed under any of the following conditions: total cholesterol levels ≥6.2 mmol/L, low‐density lipoprotein cholesterol ≥4.1 mmol/L, high‐density lipoprotein cholesterol <1.0 mmol/L, or triglycerides ≥2.3 mmol/L [51].

### 2.5. Statistical Analysis

To characterize the baseline features of both normal and diabetic patient groups, categorical variables were represented as percentages and analyzed using chi‐square tests. Continuous variables, such as age and gender, were presented as mean ± standard deviation and assessed using rank–sum tests.

Initially, multivariable logistic regression analysis was conducted to explore the associations between various metal ions in saliva and diabetes. PSM was utilized to balance all covariates included in the study, aiming to mitigate selection bias from covariates. A series of models were constructed after adjusting covariates (Table S4), with Model 1 making no covariate adjustments. Models 2, 3, 4, and model 5 were multivariable logistic regression models. Model 2 adjusted for covariates including age, gender, and education level, while model 3 included additional covariates of smoking status and alcohol use. Model 4 adjusted for all study covariates, while model 5 excluded individuals with HTN and HPL. Model PSM conducted univariate logistic regression after PSM matched all pertinent covariates. To facilitate statistical analysis and comparison, the units of saliva metal ions at different concentration levels were adjusted (Table S5).

Subsequently, a RCS analysis was conducted to evaluate the dose–response relationship between the concentrations of metal ions in saliva and the risk of diabetes. Potential nonlinear associations were assessed using *P*‐overall and *P*‐nonlinear) values.

Data extraction, analysis, and image production in this study were executed using R software (version 4.4.1, R Foundation for Statistical Computing, Austria). All statistical analyses utilized two‐sided *p*‐values, with a threshold of *p* ≤ 0.05 indicating statistical significance.

## 3. Results

### 3.1. Characterization of Study Participants

The demographic characteristics of the participants included in the study are summarized, distinguishing between those diagnosed with diabetes and those without (Table 1). Individuals with a fasting plasma glucose level ≥7.0 mmol/L or an HbA1c value ≥6.5% can be diagnosed with diabetes (the ninth edition of the Internal Medicine Diagnostic Standards). Out of 912 participants, 138 were diagnosed with diabetes. There were significant differences in age, gender, smoking status, BMI, and prevalence of HPL between the diabetic and control groups (all *p* ≤ 0.05). The analysis indicated that participants in the diabetic group were older and had higher BMIs on average, with diabetes occurring more frequently among males and individuals with HPL.

**Table 1:** Characteristic of covariates.

Level	Overall^a^	No diabetes	Diabetes	Statistic	*P* ^b^
No.	912	774	138	—	—
Age (mean [SD])	52.14	51.18	57.55	36300.5	<0.001
Gender (%) 0	453 (49.7)	362 (46.8)	91 (11.8)	16.462	<0.001
1	459 (50.3)	412 (53.2)	47 (6.1)	—	—
Education (%) 1	63 (6.9)	52 (6.7)	11 (1.4)	8.417	0.135
2	173 (19.0)	152 (19.6)	21 (2.7)	—	—
3	306 (33.6)	246 (31.8)	60 (7.8)	—	—
4	194 (21.3)	169 (21.8)	25 (3.2)	—	—
5	102 (11.2)	90 (11.6)	12 (1.6)	—	—
6	74 (8.1)	65 (8.4)	9 (1.2)	—	—
Smoking (%) 0	626 (68.6)	555 (71.7)	71 (9.2)	22.453	<0.001
1	209 (22.9)	161 (20.8)	48 (6.2)	—	—
2	77 (8.4)	58 (7.5)	19 (2.5)	—	—
Drinking (%) 0	401 (44.0)	342 (44.2)	59 (7.6)	5.717	0.057
1	337 (37.0)	294 (38.0)	43 (5.6)	—	—
2	174 (19.1)	138 (17.8)	36 (4.7)	—	—
HTN (%) 0	601 (65.9)	517 (66.8)	84 (10.9)	1.576	0.209
1	311 (34.1)	257 (33.2)	54 (7.0)	—	—
HPL (%) 0	616 (67.5)	551 (71.2)	65 (8.4)	29.908	<0.001
1	296 (32.5)	223 (28.8)	73 (9.4)	—	—
BMI (mean [SD])	24.83	24.65	25.79	41795	<0.001

Abbreviations: BMI, body mass index; HPL, hyperlipidemia; HTN, hypertension; SD, stand error.

^a^Continuous variables such as age and gender are the mean (standard deviation), and other categorical variables are the number of people (percentage).

^b^chi‐square test was used for continuous variables and rank–sum test for classified variables.

### 3.2. Comparative Analysis

**Figure 1:**
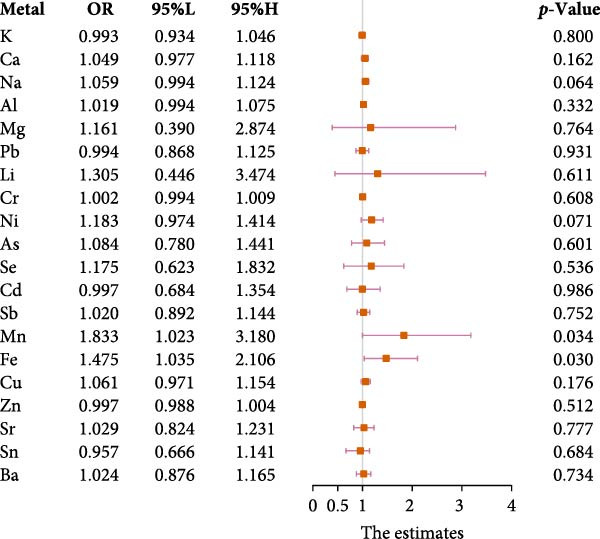
Results of the multifactorial logistic regression analysis for 20 metal ions.

In the models developed (Table S4), Model 4 matched the previously mentioned eight covariates. Following this adjustment, characteristics of the continuous variables representing metal ions in saliva were outlined in Table S6. Multivariable logistic regression was conducted using Model 4, and forest plots were generated. Salivary Mn and Fe concentrations in diabetics and controls were presented in Table S6. The results indicated that the associations between manganese (Mn) and iron (Fe) saliva and diabetes were statistically significant, with odds ratios (ORs, 95% confidence interval [CI]) of 1.833 (1.023, 3.180) and 1.475 (1.035,2.106), respectively (Figure 1). An OR > 1 indicates a positive association, while an OR < 1 suggests a negative association [52]. These findings suggested a positive association between salivary Mn and Fe levels and diabetes risk. Subsequent analyses using Model 5, after excluding patients with HPL and HTN, indicated that the relationships between saliva metal ions and diabetes lost statistical significance (*p*>0.05). This result suggests that HPL and HTN may act as confounding factors, significantly altering the statistical significance of the relationships between Mn and Fe in saliva and diabetes (Figure S1).

**Figure 2:**
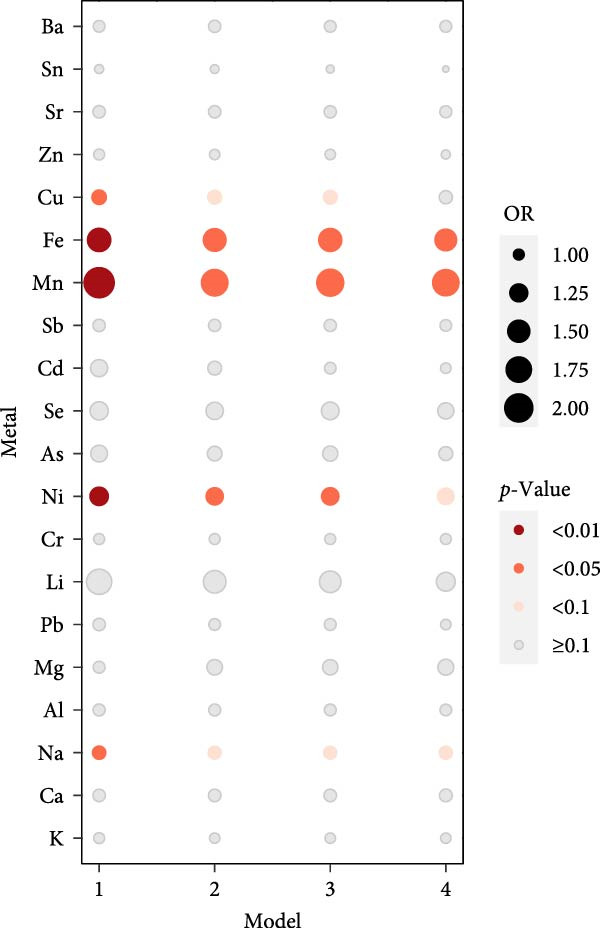
Logistic regression results of four models: Model 1 made no covariate adjustments. Model 2 adjusted for age, gender, and education; Model 3 further included smoking and alcohol use; Model 4 adjusted for all eight covariates.

Logistic regression analyses were applied to the concentrations of metal ions in saliva using four previously described models, and the results were visualized in bubble charts (Figure 2). Model 1 conducted a univariate logistic regression analysis. The associations between metal ion concentrations in saliva and diabetes risk were summarized, with circle sizes denoting ORs and colors reflecting *p*‐values (Figure 2). Across all models, Fe and Mn demonstrated a significant positive correlation with diabetes risk. Nickel (Ni) exhibited a significant positive correlation in the initial three models, which became statistically insignificant when more covariates (BMI + HPL + HTN) were added. Only in Model 1, copper (Cu) and sodium (Na) in saliva were significantly associated with diabetes risk.

### 3.3. PSM Analysis

After balancing the eight covariates of this study using Module PSM (Table S4), a univariate logistic regression analysis was conducted to assess the correlation between saliva metal ion concentrations (continuous variables) and diabetes risk. Post adjustment with PSM, it was found that concentrations of Na, Ni, Mn, Fe, and Cu in saliva significantly correlated with an increased risk of diabetes (*p* ≤ 0.05), with (ORs, 95% [CI]) of 1.062 (1.002, 1.123), 1.274 (1.072, 1.521), 2.207 (1.337, 3.647), 1.590 (1.156, 2.229), and 1.100 (1.015, 1.189), respectively (Figure S2). Similar to the previous multivariable logistic regression analyses (Figures 1,2), this demonstrates that PSM effectively mitigates the impact of confounders, ensuring the models’ stability and reliability.

### 3.4. RCS

**Figure 3:**
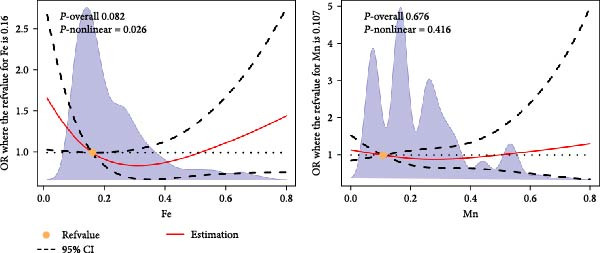
RCS results of the nonlinear dose–response relationships between salivary Fe, Mn concentrations and diabetes risk. The *x*‐axis represents the concentrations of Fe and Mn, with the units specified as mg/Kg.

Synthesizing findings from comparative and PSM analyses, significant statistical associations were identified between the concentrations of Fe and Mn in saliva and diabetes risk. The dose–response correlations between Fe, Mn and diabetes were analyzed using RCS, revealing that the concentration of Fe in saliva exhibits a complex nonlinear relationship with diabetes risk (*p*‐nonlinear ≤ 0.05, Figure 3). Concentrations of Fe below a specified reference value (0.16 mg/kg) are associated with reduced diabetes risk, whereas levels above this threshold increase the risk. No nonlinear relationship was detected between Mn concentration in saliva and diabetes risk (*p*‐nonlinear > 0.05). Additionally, subgroup RCS analysis, stratified by age and gender, demonstrated a nonlinear relationship between the concentration of Fe in male saliva and diabetes risk, aligned with the general dose–response trend (Figure S3). No nonlinear relationships were found in other subgroup analyses.

## 4. Discussion

In this study, the salivary metal ion levels of all participants were examined, and the differences between the control and diabetic groups were compared. After adjusting for all covariates using PSM, a significant association between salivary metal levels and diabetes risk was observed. This method more effectively corrected for confounding factors, such as selection bias. In addition, RCS analysis demonstrated a dose–response relationship between salivary Fe and Mn levels and diabetes, which strengthened the reliability of our results. Notably, this study was the first to identify a significant nonlinear relationship between salivary Fe levels and diabetes. The results show that salivary Fe concentrations of 0.16 mg/kg represent a critical threshold, with levels below this associated with reduced diabetes risk and levels above it linked to increased risk. This threshold has the potential to define a clinical reference range to inform diabetes screening protocols. This finding provided strong evidence for the potential use of salivary Fe concentration as a biomarker for diabetes diagnosis and monitoring. Most importantly, it serves as a reminder that regular monitoring of salivary Fe concentration can track changes overtime, reflecting disease progression or treatment effectiveness.

In this study, the salivary metal ions Fe and Mn were found to be significantly associated with diabetes and have also been implicated in other diseases. Fe, an essential trace element, plays a critical role in cellular functions [53]. Disruption of iron homeostasis generates reactive oxygen species (ROS) via the Fenton reaction, leading to lipid, protein, and DNA damage [54], and is linked to cancer, oral diseases, and neurological disorders [55, 56]. ROS produced by Fe has been shown to contribute to periodontitis by exacerbating oxidative damage to gingival tissue, periodontal ligaments, and alveolar bone [57]. Mn, an essential component of manganese superoxide dismutase (MnSOD), regulates oxidative stress and mitochondrial function [58]. Elevated Mn levels in primary teeth serve as biomarkers for neurotoxicity, reflecting early developmental exposure [59]. Increased salivary Mn also offers potential for early diagnosis of manganese toxicity [35].

Based on this study and previous research, salivary Fe and Mn are believed to be closely associated with the biological mechanisms of diabetes, including oxidative stress, inflammation, metabolic regulation, and signaling pathways [60, 61]. Furthermore, metabolic changes in the saliva of diabetic patients may impact the oral environment [62], potentially worsening systemic complications such as cardiovascular disease, nephropathy, and neuropathy through chronic inflammation and bacterial infection [3, 63].The increase in salivary Fe and Mn levels may be caused by multiple factors, including diet, environmental exposure, and disease effects. Notably, dental materials containing Fe and Mn have been shown to release metal ions into saliva [64, 65]. Thus, the potential risks should be assessed before using oral metal materials in diabetic patients. Furthermore, as additional covariates were included in the logistic regression model, the associations between some metal ions (Cu, Ni) and diabetes risk became nonsignificant. We hypothesize that the statistically significant associations observed in the unadjusted data may have been influenced by covariates.

Currently, blood, urine, and saliva are the most commonly used fluids for systemic disease detection [66]. Among these, saliva is receiving increasing attention due to its ease of collection, noninvasiveness, and resistance to coagulation [8]. These advantages make saliva particularly useful for long‐term diabetes monitoring and mechanistic studies. This study is the first to report a nonlinear dose–response relationship between salivary Fe levels and diabetes risk, highlighting its potential as a biomarker for diabetes prediction. This discovery provides a foundation for developing noninvasive and convenient saliva‐based methods for diabetes detection.

This study has certain limitations. First, a larger sample size would be needed to better characterize metal ions in the saliva of diabetic patients. Second, the metal detection methods used measure the types and concentrations of metals but do not provide detailed information about their elemental charge or oxidation states. Although a significant nonlinear association was observed between salivary Fe concentrations and diabetes risk, further research is needed to confirm its clinical utility as a biomarker.

## 5. Conclusions

This study identified significant associations between the concentrations of iron (Fe) and manganese (Mn) in saliva and diabetes risk. No nonlinear relationship was observed between manganese concentrations in saliva and diabetes. Iron concentrations within reasonable limits (< 0.16 mg/kg) are associated with a reduced incidence of diabetes, while elevated levels (≥0.16 mg/kg) may increase diabetes risk. Monitoring metal ion levels in saliva may facilitate the prevention and management of diabetes.

## Conflicts of Interest

The authors declare no conflicts of interest.

## Author Contributions

**Zihan Ding:** conceptualization, data curation, formal analysis, methodology, validation, writing – original draft preparation. **Jieyu Ming:** conceptualization, data curation, writing – review and editing. **Huajun Dai:** investigation, data curation, writing – review and editing. **Liling Chen:** data curation, formal analysis, writing – review and editing. **Bing Guo:** formal analysis, writing – review and editing. **Weiqi Li:** resources, software, writing – review and editing. **Xing Zhao:** visualization, supervision. **Hang Zhao:** conceptualization, visualization, project administration. **Hao Xu:** conceptualization, methodology, visualization, funding acquisition, writing – review and editing. Zihan Ding, Jieyu Ming, Huajun Dai and Liling Chen contributed equally to this work.

## Funding

This work was supported by the research funding from the West China School/Hospital of Stomatology, Sichuan University (Grant RCDWJS2023‐21) and the National Natural Science Foundation of China (Grants 82103943 and 81973151).

## Supporting Information

Additional supporting information can be found online in the Supporting Information section.

## Supporting information


**Supporting Information** The Supporting Information submitted along with our manuscript comprise six tables and three figures. The specific titles of the tables and figures are as follows: Table S1. Working parameters of microwave digestion. Table S2. Working parameters of ICP‐MS. Table S3. Working parameters of ICP‐OES. Table S4. Details of the adjusted covariates for each model. Table S5. Details on the adjustment of metal units. Table S6. Characteristics of metal ions. Figure S1. Results of the logistic regression analysis using model 5. Figure S2. Results of the univariate logistic regression analysis using model PSM. Figure S3. RCS results of the nonlinear dose–response relationships between Fe, Mn concentrations and diabetes risk after stratification by age and gender.

## Data Availability

All data generated or analyzed during this study are included in this article and its Supporting Information. Tables S1–S6 and Figures S1–S3 are provided in the Supporting Information. Further enquiries can be directed to the corresponding authors.
