# Supplementary material for: Salivary Metal Ions as Potential Biomarkers for Diabetes: An Observational Study
Source: Int J Dent. 2026 Jan 16;2026:9224685. doi: 10.1155/ijod/9224685 (PMC12809914; doi:10.1155/ijod/9224685)
Supplement: Supplementary file 1 — Supporting Information The Supporting Information submitted along with our manuscript comprise six tables and three figures. The specific titles of the tables and figures are as follows: Table S1. Working parameters of microwave digestion. Table S2. Working parameters of ICP‐MS. Table S3. Working parameters of ICP‐OES. Table S4. Details of the adjusted covariates for each model. Table S5. Details on the adjustment of metal units. Table S6. Characteristics of metal ions. Figure S1. Results of the logistic regression analysis using model 5. Figure S2. Results of the univariate logistic regression analysis using model PSM. Figure S3. RCS results of the nonlinear dose–response relationships between Fe, Mn concentrations and diabetes risk after stratification by age and gender. [file IJOD-2026-9224685-s001.docx]

Supplementary Materials for

**Salivary metal ions as potential biomarkers for diabetes: an observational study**

Zihan Ding ^a,#^, Jieyu Ming ^a,#^, Huajun Dai ^b,#^, Liling Chen ^c,#^, Bing Guo ^d^, Weiqi Li ^a^, Xing Zhao ^d,*^, Hang Zhao ^a,*^, Hao Xu ^a,*^

^a^ State Key Laboratory of Oral Diseases & National Center for Stomatology & National Clinical Research Center for Oral Diseases & Research Unit of Oral Carcinogenesis and Management & Chinese Academy of Medical Sciences, West China Hospital of Stomatology, Sichuan University, Chengdu 610041, Sichuan, China

^b^ Chengdu Institute for Food and Drug Control, Chengdu 610041, Sichuan, China

^c^ Chongqing Municipal Center for Disease Control and Prevention, Chongqing Municipality 402100, China

^d^ West China School of Public Health and West China Fourth Hospital, Sichuan University, Chengdu 610041, Sichuan, China

^#^ These authors contribute equally to this work.

^*^ These authors are the corresponding authors.

Corresponding E-mail: [hao.xu@scu.edu.cn](mailto:hao.xu@scu.edu.cn);

zhaohangahy@scu.edu.cn;

[xingzhao@scu.edu.cn](mailto:xingzhao@scu.edu.cn)

Other E-mails: [dzh2022@stu.scu.edu.cn;](mailto:dzh2022@stu.scu.edu.cn;)

Jennie_ming1002@163.com;

[daihuajun4504@163.com](mailto:daihuajun4504@163.com);

[mbcllgz@163.com;](mailto:mbcllgz@163.com;)

[guobing0111@foxmail.com;](mailto:guobing0111@foxmail.com;)

liweiqi@stu.scu.edu.cn

**Table S1.** Working parameters of microwave digestion.

| **Step** | **Temperature (°C)** | **Ramp time (min)** | **Hold time (min)** |
| --- | --- | --- | --- |
| 1 | 120 | 5 | 5 |
| 2 | 150 | 5 | 10 |
| 3 | 190 | 5 | 20 |

**Table S2.** Working parameters of ICP-MS.

| **Items** | **Working parameters** |
| --- | --- |
| Frequency (W) | 1550 |
| Sampler type | Nickel |
| Sample depth (mm) | 5 |
| Peristaltic pump speed (r/min) | 40 |
| Carrier gas flow rate (L/min) | 1.022 |
| Cooling gas flow rate (L/min) | 15 |
| Collision mode | Helium |
| Sample collection frequency | 3 |
| Temperature (°C) | 2 |
| Analysis mode | Full spectrum analysis |

**Table S3.** Working parameters of ICP-OES.

| **Items** | **Working parameters** |
| --- | --- |
| Sample flow rate (L/min) | 15 |
| Sample injection speed (mL/min) | 1.5 |
| Sample frequency (W) | 1300 |
| Nebulizer flow rate (L/min) | 0.55 |
| Auxiliary gas flow rate (L/min) | 0.2 |

**Table S4.** Details of the adjusted covariates for each model.

| **Model** | **Method** | **No.** | **Formula** |
| --- | --- | --- | --- |
| Model 1 | Logistic | 912 (138 diabetes) | Glm (PD~ Metal) |
| Model 2 | Logistic | 912 (138 diabetes) | Glm (PD~ Metal + Age + Gender + Education level) |

**Table S4.** Details of the adjusted covariates for each model.

| **Model** | **Method** | **No.** | **Formula** |
| --- | --- | --- | --- |
| Model 3 | Logistic | 912 (138 diabetes) | Glm (PD~ Metal + Age+ Gender + Education level + smoking status + alcohol use) |
| Model 4 | Logistic | 912 (138 diabetes) | Glm (PD~ Metal + Age+ Gender+Education level + smoking status + alcohol use + BMI+ hypertension + hyperlipidemia) |
| Model 5 | Logistic | 425 (39 diabetes) ^*^ | Glm (PD~ Metal + Age+ Gender + Education level + smoking status + alcohol use + BMI) |
| Model PSM | PSM | 274 (137 diabetes) | PSM (PD~Age + Gender + Education level + smoking status + alcohol use + BMI + hypertension+ hyperlipidemia) + Glm (PD~ Metal) |

*Omit hypertension and hyperlipidemia participants.

**Table S5.** Details on the adjustment of metal units.

| **Metal** | **unadjusted** | **adjusted** |
| --- | --- | --- |
| K | 1 | /100 |
| Ca | 1 | /10 |
| Na | 1 | /100 |
| Al | 1 | *100 |
| Mg | 1 | /10 |
| Pb | 1 | *1000 |
| Li | 1 | *100 |
| Cr | 1 | *10000 |
| Ni | 1 | *100 |
| As | 1 | *1000 |
| Se | 1 | *100 |
| Cd | 1 | *1000 |
| Sb | 1 | *10000 |
| Mn | 1 | *10 |
| Fe | 1 | *1 |
| Cu | 1 | *100 |
| Zn | 1 | *10 |
| Sr | 1 | *10 |

**Table S5.** Details on the adjustment of metal units.

| **Metal** | **unadjusted** | **adjusted** |
| --- | --- | --- |
| Sn | 1 | *100 |
| Ba | 1 | *100 |

**Table S6.** Characteristics of metal ions.

| **Metal** | **Overall^a^** | **No diabetes** | **Diabetes** | ***P*^b^** |  |
| --- | --- | --- | --- | --- | --- |
| Ca | 5.02 [ 1.43, 9.32 ] | 4.91 [ 1.73, 9.23 ] | 5.57 [ 0.3, 9.58 ] | 0.025 |  |
| Na | 2.8 [ 0.87, 7.97 ] | 2.74 [ 0.88, 7.51 ] | 2.94 [ 0.71, 8.81 ] | 0.144 |  |
| Al | 0 [ 0, 0 ] | 0 [ 0, 0 ] | 0 [ 0, 0 ] | 0.112 |  |
| Mg | 0 [ 0, 0.46 ] | 0 [ 0, 0.46 ] | 0 [ 0, 0.54 ] | 0.425 |  |
| Pb | 1.05 [ 0.23, 3.93 ] | 1.04 [ 0.23, 3.92 ] | 1.18 [ 0.37, 3.74 ] | 0.025 |  |
| Li | 0.14 [ 0.02, 0.55 ] | 0.14 [ 0.02, 0.5 ] | 0.15 [ 0.03, 0.65 ] | 0.455 |  |
| Cr | 6.79 [1.13, 36.07 ] | 6.6 [ 1.14, 34.14 ] | 8.08 [ 1.12, 76.37 ] | 0.257 |  |
| Ni | 0.31 [ 0.04, 1.96 ] | 0.31 [ 0.04, 1.67 ] | 0.36 [ 0.04, 3.23 ] | 0.114 |  |
| As | 0.35 [ 0.04, 1.41 ] | 0.35 [ 0.04, 1.38 ] | 0.35 [ 0.03, 1.59 ] | 0.450 |  |
| Se | 0.17 [ 0, 0.75 ] | 0.17 [ 0, 0.72 ] | 0.18 [ 0, 0.96 ] | 0.088 |  |
| Cd | 0.02 [ 0, 0.79 ] | 0.01 [ 0, 0.73 ] | 0.05 [ 0, 1.42 ] | 0.067 |  |
| Sb | 0 [ 0, 2.41 ] | 0 [ 0, 2.31 ] | 0 [ 0, 2.90 ] | 0.885 |  |
| Mn | 0.11 [ 0, 0.64 ] | 0.11 [ 0, 0.55 ] | 0.17 [ 0, 1.07 ] | 0.046 |  |
| Fe | 0.17 [ 0.02, 0.95 ] | 0.17 [ 0.03, 0.84 ] | 0.18 [ 0.02, 1.23 ] | 0.107 |  |
| Cu | 1.05 [ 0, 5.17 ] | 1.05 [ 0, 4.28 ] | 1.06 [ 0, 5.87 ] | 0.026 |  |
| Zn | 1.23 [ 0.31, 5.15 ] | 1.19 [ 0.31, 4.98 ] | 1.33 [ 0.31, 5.74 ] | 0.523 |  |
| Sr | 0.21 [ 0, 2.00 ] | 0.21 [ 0, 1.85 ] | 0.21 [ 0, 2.17 ] | 0.261 |  |
| Sn | 0 [ 0, 0 ] | 0 [ 0, 0 ] | 0 [ 0, 0 ] | 0.826 |  |
| Ba | 0 [ 0, 2.63 ] | 0 [ 0, 2.61 ] | 0 [ 0, 2.66 ] | 0.227 |  |

a: Median [quartile (5%,95%)] of metal; b: Rank sum test was used for all classified variables.


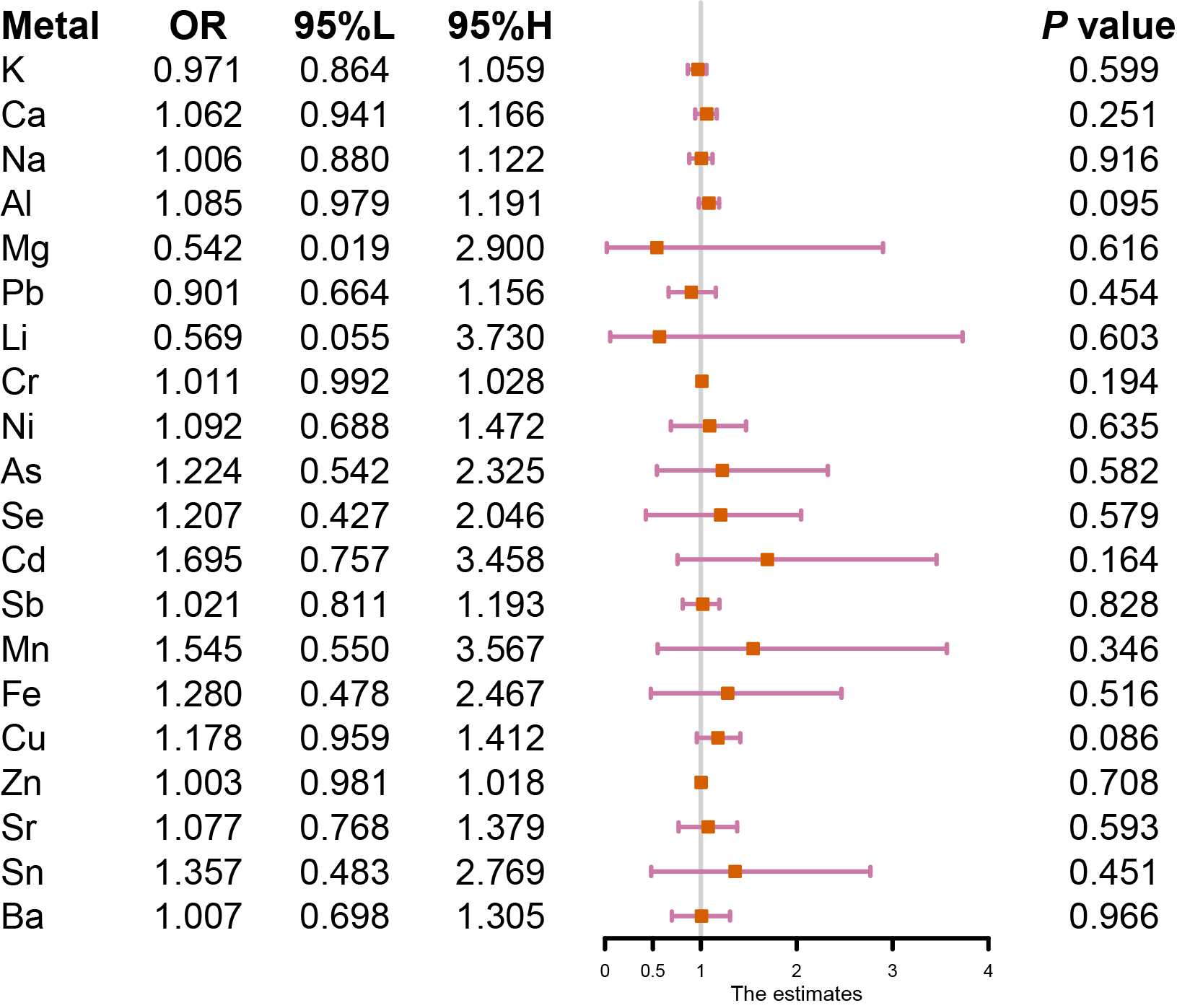


**Figure S1.** Results of the logistic regression analysis using model 5.


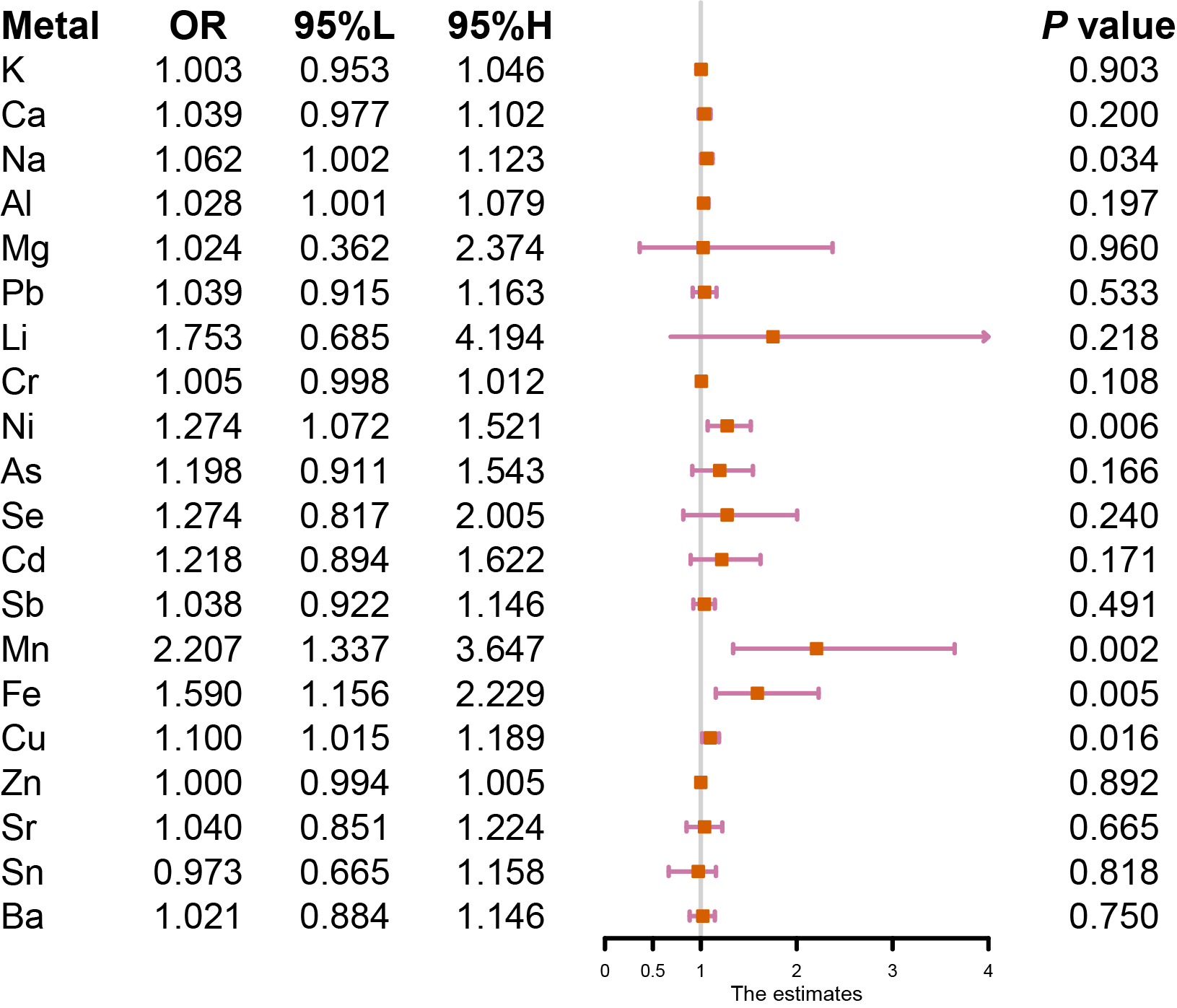


**Figure S2.** Results of the univariate logistic regression analysis using model PSM.


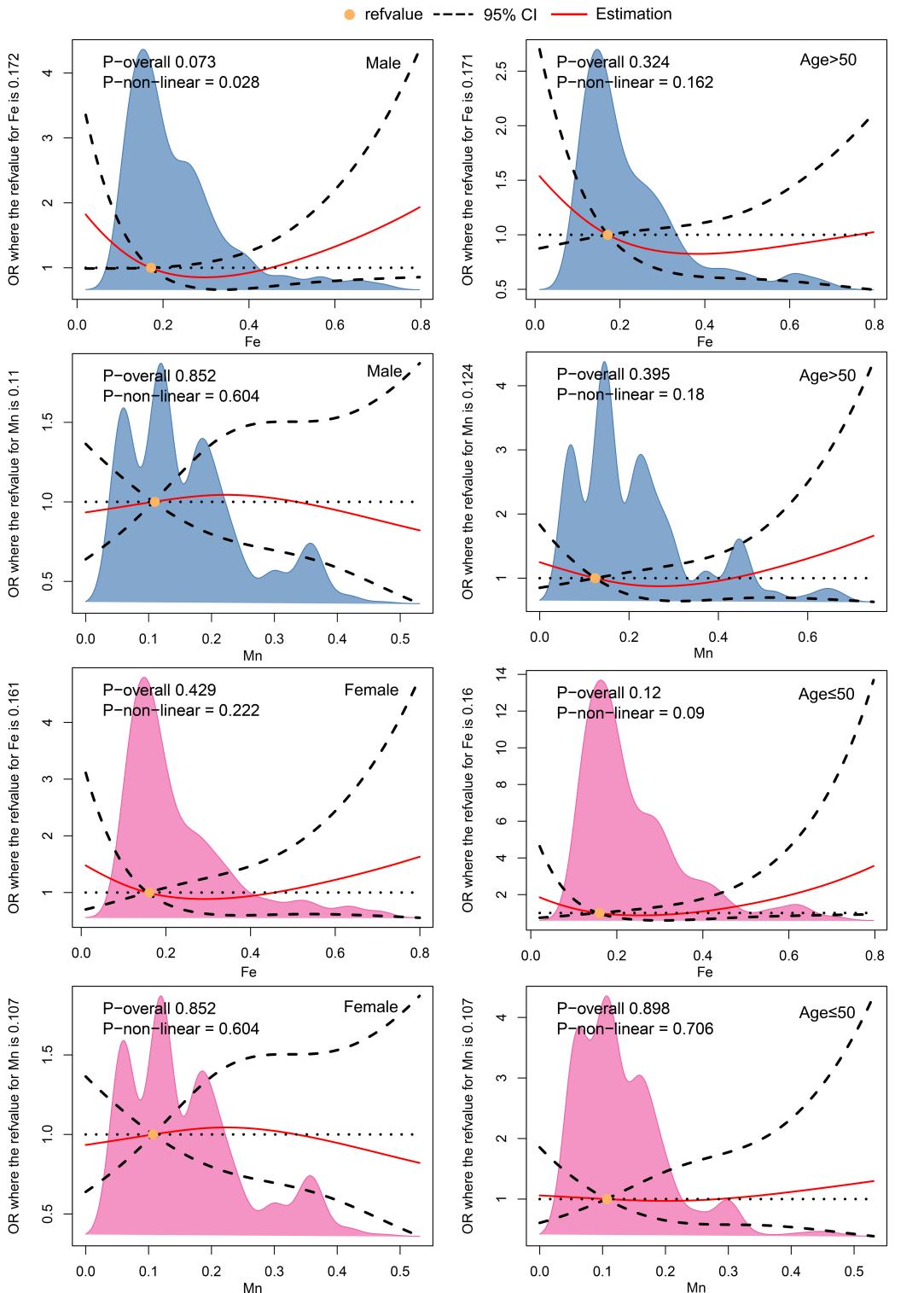


**Figure S3.** RCS results of the nonlinear dose-response relationships between Fe, Mn concentrations and diabetes risk after stratification by age and gender.
